# Major Depression in Comorbidity with Substance use Disorders: Patients’ Features and Clinical-Neurobiological Rationale of Antidepressant Treatments

**DOI:** 10.2174/1570159X22666240827165327

**Published:** 2024-08-30

**Authors:** Sergio De Filippis, Giovanni Martinotti, Ferdinando Nicoletti, Andrea Mastrostefano, Giada Trovini, Anna Pugliese, Marco Di Nicola

**Affiliations:** 1 Villa Von Siebenthal Clinic, Rome, Italy;; 2 Department of Neuroscience, Imaging, and Clinical Sciences, University G. D’Annunzio, Chieti, Italy;; 3 Department of Physiology and Pharmacology, Sapienza University, Rome, Italy;; 4 Department of Molecular Pathology, IRCCS Neuromed, Pozzilli, Italy;; 5 Medical Department, Lundbeck Italia, Milan, Italy;; 6 Department of Psychiatry, Fondazione Policlinico Universitario Agostino Gemelli IRCCS, Rome, Italy;; 7 Department of Neuroscience, Section of Psychiatry, Università Cattolica del Sacro Cuore, Rome, Italy

**Keywords:** Major depressive disorder, substance use disorder, alcohol use disorder, anhedonia, cognition, antidepressants, personalized treatment, precision psychiatry

## Abstract

The frequent co-occurrence of major depressive disorder (MDD) and substance use disorders (SUDs) entails significant clinical challenges. Compared to patients with MDD alone, patients with MDD and SUD often show increased anhedonia, emotional blunting, and impaired cognitive function. These symptoms lead to an inability to control cravings, more substance use, increased relapse rates, and poor adherence to the treatment. This fosters a detrimental cycle leading to more severe depressive symptoms, functional impairment, and chronicity, culminating in heightened morbidity, mortality, and healthcare resource utilization. Data on antidepressant treatment of MDD-SUD patients are inconclusive and often conflicting because of a number of confounding factors in clinical trials or difficulty in dissecting the specific contributions of pharmacological *versus* psychological interventions in real-world studies. The patient's unique clinical features and specific SUD and MDD subtypes must be considered when choosing treatments. Ideally, drug treatment for MDD-SUD should act on both conditions and address core symptoms such as anhedonia, craving, and cognitive dysfunction while ensuring minimal emotional blunting, absence of drug interactions, and no addictive potential. This approach aims to address unmet needs and optimize the outcomes in a clinical population often underrepresented in treatment paradigms.

## 1 INTRODUCTION

The co-occurrence of substance use disorder (SUD) and other psychiatric disorders, such as schizophrenia, bipolar disorders, and major depressive disorder (MDD), is quite common in psychiatric settings [1-4]. MDD and SUDs represent growing issues in terms of incidence and severity. The World Health Organization (WHO) estimates that more than 300 million people worldwide suffer from depression, and by 2030, MDD will be the leading cause of disability worldwide [5]. MDD is comorbid with SUD at a rate suggesting a strong association between these two conditions [6] (Table **1** for diagnostic criteria). Alcohol use disorder (AUD) is the most common of all SUDs associated with MDD; one in five subjects with MDD is likely to have AUD in their lifetime, and most people diagnosed with MDD and comorbid SUD consume alcohol [6].

In clinical practice, distinguishing between depressive syndromes occurring independently of, or within the context of, SUD is not always straightforward. Also, in MDD-SUD patients, establishing the direction of comorbidity is often challenging; does substance use precipitate the emergence of depressive symptoms, or is substance use a consequence of attempts to self-medicate an underlying depressive condition? Another question is whether MDD and MDD-SUD are distinct entities that require a different treatment approach [7, 8]. The DSM-5 distinguishes between primary MDD co-occurring with SUD and depressive disorders that are causally associated with substance use (Substance-Induced Depressive Disorder or SIDD). This differentiation is based on the temporal relationship between the onset of depression and SUD, *i.e*., whether depressive symptoms occur prior to the onset of SUD or during prolonged abstinence [6, 8]. However, this classification is a matter of debate [8]. Some studies suggest that individuals with co-occurring MDD and AUD have demographic and clinical characteristics that differ from those with SIDD, *e.g*., a greater prevalence in females, white, and married, more lifetime attempts of suicide, earlier onset of depression, and longer depressive episodes [9], higher rates of family history of mood disorders, and less propensity to seek treatment for AUD [10]. In contrast, other studies have found no significant differences between these groups and some depressive episodes initially diagnosed as SIDD were later reclassified as MDD [11-13]. Regardless of the diagnosis, the presence of a clinically significant mood disorder has negative prognostic implications for the outcome of SUD and *vice-versa* [1, 8]. Individuals with MDD-SUD experience more severe depressive symptoms, greater functional impairment, a more chronic course of illness, lower rates of recovery, higher rates of hospitalization, and increased morbidity and mortality compared to those with a single disorder [1, 6, 8, 14]. Substance use is also likely to undermine treatment efficacy, resulting in depressive relapses and increased risk of suicide attempts [6, 14-16]. Therefore, it is crucial to gain a deeper understanding of factors contributing to the MDD-SUD comorbidity and develop interventions that can target both MDD and SUD simultaneously. Indeed, a dual approach has been shown to improve symptoms, reduce relapse rates, promote recovery, and achieve better overall outcomes [17-23].

Here, we review the epidemiology and clinical characteristics of individuals with MDD and comorbid SUD, as well as the efficacy, effectiveness, and neurobiological rationale of different antidepressant treatments in targeting both MDD and SUD symptoms. We focused on clinical features particularly evident in (and possibly underlying) both conditions (*e.g*., anhedonia, emotional blunting, and cognitive symptoms) and how these are affected by pharmacological treatment. Because alcohol is the most commonly used substance worldwide [24], and AUD is the most widespread among SUD [6], we addressed MDD-SUDs and MDD-AUD separately.

## 2 EPIDEMIOLOGY OF MDD-SUD

The prevalence of co-occurring MDD-SUD varies between 12% and 80% across different studies. This wide range is explained by several factors, including whether the study was conducted with a sample of substance users or among the general population, whether participants were recruited in addiction treatment centers, mental health care facilities, or other settings (prisoners, homeless), methodological aspects (like diagnostic criteria, use of either DSM or ICD, in their various versions), the main substance consumed (tobacco, alcohol, cocaine, opiates, sedative-hypnotics, others), *etc*. [25, 26].

A close link between MDD and SUD emerged from a meta-analysis of epidemiological studies including >500,000 subjects in the general population between 1990 and 2014 [27]. A more recent meta-analysis including almost 350,000 subjects showed high rates of SUDs in patients with major depression (0.250 prevalence rate) [6]. The association of MDD with AUD was the most common (prevalence rate: 0.208; 95% CI 0.18-0.24) and was higher than that with illicit substances (prevalence rate: 0.12; 95% CI 0.09-0.15) [6]. It should be noted that the meta-analysis by Hunt *et al*. [6] only partially reflects the current situation, also considering the recent surge in opioid - namely fentanyl - and methamphetamine use in North America and cannabis potency and use worldwide [28-31]. A more recent meta-analysis including >100,000 patients with opioid use disorder (OUD) found depression to be the most prevalent psychiatric disorder in this population (especially in women), with a prevalence rate of 36.1% (95% CI: 32.4-39.7%) [32]. This is of great relevance in North America, where the increase in fentanyl use has grown to epidemic proportions [30].

The rate of cannabis use in subjects affected by depression is more than three times higher than in the general population (11.7% *vs.* 3.8%, respectively) [6, 33]. One epidemiological study carried out in the Netherlands found that nearly 30% of patients affected by MDD used cannabis at very high rates [34]. The use of cannabis was already legal in the Netherlands at that time. Owing to the dramatic rise in cannabis use [16], the association between cannabis use and high odds of depression and depression severity is now spread worldwide, particularly in adolescence [35, 36].

Depression is also common in individuals affected by psychostimulant use disorder, which may develop as a result of psychostimulant-induced monoamine depletion and neurotoxicity [37-42].

### 2.1 Epidemiology of MDD-AUD

The lifetime prevalence of AUD in MDD has been reported to be as high as 40% [43, 44]. In the meta-analysis by Lai *et al*. [27] (not based on DSM-V criteria), the pooled OR for major depression and alcohol use was 1.53, and the association was strongest for alcohol dependence (OR 3.094). Hunt *et al*. [6] found a prevalence rate of 0.208 (with no difference between patients with MDD and those with persistent depressive disorder), consistent with previous data reporting that one in five patients with MDD is likely to develop AUD in his/her lifetime [14, 15, 45]. The pooled prevalence of AUD and MDD is balanced in the two sexes because of the higher prevalence of AUD in males and the higher prevalence of depression in females. This differs from the pooled prevalence of AUD with schizophrenia or bipolar disorder, which is higher in males [2, 3]. Finally, despite the high prevalence and the increased incidence during the pandemic, AUD is sometimes underdiagnosed, especially in cases of binge drinking behaviors [46, 47]. While the prevalence of these risky conducts has been spreading over the last years, also in association with multiple substance use, their short- and long-term effects on depressive spectrum disorders are still underestimated.

## 3 CLINICAL CHARACTERISTICS OF MDD-SUD COMORBIDITY *VS.* MDD

Patients with comorbidity between MDD and SUD show different clinical features than those with MDD alone [8] (Table **2**). The co-occurrence of depression and SUD is a complex and multifaceted issue in mental health, and overlapping risk factors for both diagnoses have been proposed, such as a family history of mental illness [8]. Differences between MDD with and without SUD were investigated in a nationally representative sample of US adults of over 43,000 subjects [8]. This study provided interesting insights, such as a lifetime prevalence of MDD and MDD-SUD of 7.41% and 5.82%, respectively, and more severe depressive symptoms in individuals with MDD-SUD compared to those with MDD alone [8, 48] (Table **2**). The reverse is also true, as suggested by the evidence that cocaine users with depression experience greater euphoria in response to cocaine [49]. This may generate a vicious cycle between psychostimulant use and mood instability.

Chronic use of cannabis is associated with an increased risk of developing depressive disorder [35, 50-53]. A meta-analysis including >23,000 individuals found an OR of depression in young adult cannabis users of 1.37 (95% CI, 1.16-1.62). The pooled ORs for suicidal ideation and attempts were 1.50 (95% CI, 1.11-2.03) and 3.46 (95% CI, 1.53-7.84), respectively [35]. A recent study carried out on>6.6 million Danish individuals found that cannabis use significantly increases the risk of both psychotic and non-psychotic unipolar depression [52]. A higher risk for suicidal ideation, plan, and attempt has also been reported in cannabis users with and without a history of MDD, with an increasing trend in recent years [53]. Thus, although individual-level risk remains moderate-to-low, the high prevalence of cannabis consumption among adolescents will likely result in a large number of young people who might develop depression and suicidality attributable to cannabis [35].

OUD is more severe in individuals with comorbid psychiatric disorders in terms of physical health issues, psycho-social health problems, poor OUD treatment outcomes, morbidity, and mortality [32, 54-58]. In addition, comorbidity with psychiatric disorders limits patients’ adherence to OUD treatments, including opioid agonist therapy (OAT), which is protective in these patients [32, 59]. However, it remains unclear how MDD comorbidity and antidepressant treatment may influence OAT initiation and discontinuation [60] (see also section 4. Antidepressant treatment of MDD-SUD). This aspect is of great relevance because the lack of exposure to OAT can induce a vicious circle of adverse health outcomes and exacerbate psycho-social problems [61-63]. Some authors report that mental disorders either increase [64, 65] or have no effect [66] on OAT retention; conversely, Friesen *et al*. [67] found a ~60% increase in the risk of premature OAT discontinuation in OUD patients with psychiatric comorbidities. Buprenorphine is underutilized and often discontinued among patients affected by OUD with co-occurring mood and other psychiatric disorders [60]. It should be highlighted that the impact of comorbid psychiatric disorders on OAT depends on the specific opioid agonist and treatment schedule [58]. Accordingly, adherence to buprenorphine treatment was significantly reduced in patients with OUD and comorbid psychiatric disorders, whereas methadone treatment was unaffected. On top of all these issues, the surge in fentanyl use has majorly impacted the clinical practice and is a huge barrier to the successful treatment of SUD and psychiatric comorbidities, as many past SUD treatment methods no longer work as well as they used to. The lack of data on how fentanyl has impacted the management of these patients warrants further research in this setting.

Interestingly, in individuals with MDD-SUD comorbidity, substance use is enhanced in the year preceding antidepressant medication [68]. This is of particular relevance because the rate of treatment-resistant depression is greater when antidepressants are combined with heavy consume of substances, such as sedatives, opioids, alcohol, alone or in combination [69].

Finally, patients with MDD-SUD are less likely to receive drug treatment for depression compared to those with MDD alone [70]. It is estimated that only 10% of AUD-MDD patients seek medication in Europe, and more than two-thirds relapse within the first 12 months of treatment [47, 71-73]. In the US, less than 60% of MDD-SUD-diagnosed individuals received any treatment for depression [74]. Coughlin *et al*. [70] found that patients with comorbid MDD-SUD had ~20% lower odds of guideline-concordant acute pharmacological treatment, ~25% lower odds of drug treatment continuation, ~15% lower odds of adequate acute-phase psychotherapy, and ~20% lower odds of psychotherapy continuation. These rates resemble those reported by other studies showing reductions in any guideline-concordant depression treatment in patients with comorbid SUD [74, 75]. Nevertheless, there is evidence that high-quality depression care, including antidepressant medication, is effective in treating depressive symptoms across different SUDs and may even improve SUD itself [22, 23, 68, 76-79]. However, the effects of antidepressant treatment on the outcome of SUD are still debated and often transient (see section 5. Antidepressant treatment of MDD-SUD comorbidity).

### 3.1 Focus on Patients with MDD *vs.* MDD-AUD

Patients with comorbid MDD-AUD show a more severe course of illness, serious cognitive impairments, worse adherence and response to treatment, and a higher risk of suicide [80, 81].

A family history of AUD, childhood trauma, psychotic features, previous suicide attempts, and panic disorder are frequently found in MDD-AUD [82, 83]. Alcohol and nicotine addiction show significant genetic correlations with different psychiatric disorders, including MDD [84]. Genetics may play a role in the comorbidity of MDD and AUD, predisposing individuals with an elevated polygenic risk for MDD to develop AUD [85]. Mood disorders may also impact the transition across the various stages of alcoholism and hinder remission, particularly in women [86]. A heavier consume of alcohol is associated with a more severe depression, especially in females, although AUD is more frequent in males [87]. Accordingly, AUD severity (calculated on the basis of DSM-5 AUD criteria) was a significant linear predictor of a first-incident depressive disorder after a 3-year follow-up (0 *vs.* all DSM-5 AUD criteria = 4.20% *vs.* 44.47%, respectively) [88]. Changes in body weight or appetite, sadness and anhedonia, guilt, reduced concentration, indecisiveness, thoughts of death, and sleep disorders are associated with alcohol consume, dependence, or both [83]. Insomnia, which is prevalent in AUD and affects 60-70% of patients [89], is a robust predictor of relapse [89-91], future suicide attempts [92], and depression [93, 94]. Moreover, insomnia tends to persist despite treatment [95-97], and might pave the way for other addictive comorbidities, such as cocaine and heroin use disorder [98]. In a study by Kolla *et al*. [99], baseline sleep disturbances were significantly associated with depressive and anxious symptoms, alcohol craving when experiencing unpleasant emotions, physical discomfort, loss of personal control, conflict, number of drinks, drinking days, and hazardous drinking days.

### 3.2 Focus on Specific Clinical Features of MDD-SUD

#### 3.2.1 Anhedonia

The US National Institute of Mental Health has recognized anhedonia, *i.e*., reduced interest or enjoyment in response to rewarding stimuli, as a “significant Research Domain Criterion”. Anhedonia is an established component of both SUD and MDD and may highly contribute to the comorbidity of the two disorders [100]. Anhedonia, an established hallmark of MDD, may be considered as a trait and a state dimension, acting as a vulnerability factor for the development of SUD [100-102]. In addition, anhedonia may also develop as a result of chronic alcohol or substance use [103]; hence, it has been hypothesized that anhedonia may be a linking bridge between SUD and MDD [100].

Recent data suggest the existence of subtypes of depression with anhedonia as the predominant feature, including an “inflammatory” subtype, in which neuroinflammation might be considered as a potential *trait d’union* between early life stress and anhedonia [104]. Knowing that early life experiences shape the vulnerability to developing SUD later in life [105, 106], it can be predicted that the inflammatory subtype of depression has a high comorbidity with SUD.

Anhedonia has a negative impact on the course and treatment outcomes of SUD, being associated with craving, higher rates of relapse, and shorter periods of post-treatment abstinence [100, 107]. Similarly, in depression, anhedonia has a negative impact on progression and response to treatment [108-111].

Drug treatment of anhedonia in depression is still suboptimal. Selective serotonin reuptake inhibitors (SSRIs) showed a limited benefit and may even exacerbate anhedonic symptoms and induce motivational syndrome in some patients [112]. Treatments that were proven to improve anhedonia include agomelatine, acetyl-L-carnitine, bupropion, vortioxetine, esketamine, transcranial magnetic stimulation, cognitive-behavioral therapy, and behavioral activation [113-125]. Intranasal esketamine has shown a remarkable effect on anhedonia, even in cases of severe, resistant depression comorbid with SUDs [113-118]. Remarkably, vortioxetine has both short- and long-term therapeutic efficacy on anhedonia. Pooled analyses including almost 5,000 MDD patients showed a greater effect on anhedonia of vortioxetine with respect to placebo and to agomelatine on 500 patients [119]; improvement of anhedonia with vortioxetine was maintained for at least one year [126].

#### 3.2.2 Emotional Blunting

Emotional blunting is characterized by reduced responsiveness, low motivation, apathy, and a general emotional restriction, which results in emotional indifference and detachment [112, 127]. Abnormalities in emotion regulation are common among individuals with AUD and SUD [128, 129]. Interestingly, there are gender differences in brain responsiveness to emotional stimuli in AUD, with abstinent alcoholic women showing greater brain responses and men having lower brain responses with respect to non-alcoholic controls [130]. Emotional blunting may develop as a consequence of substance use or withdrawal. However, the evidence that the neural processing of negative affective stimuli is similar between individuals affected by SUD and internet gaming disorder [131] suggests that emotional dysregulation is a core feature of addiction.

About 50-60% of patients affected by MDD under treatment with SSRIs or serotonin-noradrenaline reuptake inhibitors (SNRIs) show some degree of emotional blunting, which may be related to medication rather than MDD [132-134]. Vortioxetine was shown to reverse emotional blunting in patients affected by MDD who were under treatment with SSRIs or SNRIs [135]. Drugs that improve emotional blunting might be valuable in individuals with comorbid MDD and SUD.

#### 3.2.3 Cognitive Symptoms

Cognitive dysfunction lies at the core of both MDD and SUD owing to the functional connection of brain networks underlying cognitive functions and processing of salience, reward, and motivation [136-140].

Cognitive dysfunction in MDD should not be confounded with the “negative cognitive bias” (*i.e*., the propensity of patients to process negative emotional information) but refers to abnormalities in executive functions, speed of processing, attention, and learning, and memory, which may outlast the improvement of mood induced by antidepressants or other treatment strategies [141-143]. SUD is frequently associated with impairment in attention, memory, and executive function. For example, 30-80% of individuals with AUD and 30-50% of individuals with psychostimulant use disorder show some degree of cognitive impairment [144]. Cognitive deficits in SUD are not only related to drug-associated cues but also extend to non-drug reinforcement tasks. In addition, deficits in executive functions are associated with negative clinical outcomes, including early relapses and poor adherence to treatment. Substance-induced maladaptive neuroplasticity can exacerbate pre-existing cognitive deficits of events that promote cognitive dysfunction. This process underlies the long-lasting vulnerability to relapse, which outlasts the resolution of the withdrawal syndrome in individuals affected by SUD [144].

Not surprisingly, cognitive dysfunction has been identified by the FDA as a target for pharmacological treatments in patients with MDD [145], and drugs effective in improving cognition may induce full functional recovery in these patients [146, 147]. Similarly, in SUD, an improvement in cognitive function is associated with lower relapse rates and better clinical outcomes [144]. Hence, antidepressants that enhance cognitive functions might be valuable in the treatment of MDD-SUD. In a network analysis of studies using the digit symbol substitution test, vortioxetine showed superiority with respect to all other antidepressants or classes of antidepressants in improving cognitive function [101]. The pro-cognitive activity of vortioxetine in the treatment of MDD is well established [102, 121, 135, 141, 148-151]. There is also evidence for a pro-cognitive action of vortioxetine in patients affected by MDD-SUD [152].

#### 3.2.4 Traumatic Load

Individuals who experience childhood traumas are at higher risk for AUD and SUD [153-157]. Childhood adversities, including physical and sexual abuse, are also associated with depression and other psychiatric disorders [155, 158-161]. We have already discussed the link between early life stress, inflammation, anhedonia, and MDD-SUD [8, 82, 100, 105, 106]. In the Adverse Childhood Experiences Study, including over 17,000 adult participants, individuals who reported three or more types of childhood traumas showed a 3- and 5-fold greater risk of developing MDD and SUD, respectively [155]. The link between early life stress and depression/SUD later in life is fully confirmed in preclinical studies. In rodents, the offspring of dams exposed to gestational stress showed an increased depressive-like behavior and cocaine or alcohol preference later in life [162-164].

#### 3.2.5 Risk of Death

Association between MDD and SUD increases the risk of suicidal behavior and premature death: a population-based cohort study including over 7,500,000 subjects living in Denmark over a 22-year period showed a 3-fold increase in mortality in individuals with mood disorders associated with SUD [1]. SUD alone and comorbid SUD-MDD and AUD-MDD are also associated with a higher risk of suicide, with the risk being positively correlated with the extent of alcohol consumption [165-168].

#### 3.2.6 Polysubstance Use

Polysubstance use complicates the management and worsens the clinical outcome of individuals affected by SUD and comorbid depression. This phenomenon is frequently described in individuals affected by OUD but is now common in all types of SUD. Polysubstance users affected by SUD have an increased incidence of anxiety and depression [169-175]. War veterans who used multiple substances were more likely to be homeless and suffer from hepatic diseases and psychiatric conditions, including MDD; in addition, they had more prescriptions of multiple psychotropic medications, greater use of psychiatric inpatient care, and residential and rehabilitative treatment [176].

It should be noted that previous research on polysubstance use is somewhat limited by the lack of “polysubstance use” definition. Recent attempts identified three key concepts necessary to define polysubstance use, namely (1) substances involved (number and type of substance use, presence of one or more SUDs, and primary and secondary substance use), (2) timing (whether simultaneous, sequential, and same-day polysubstance use, short- and long-term behavior, *etc*.), and (3) intent (namely what are the motivations and if substance use is intentional). Employing consistent definitions of polysubstance use can facilitate the integration of evidence, contributing to more effective strategies in SUD treatment [177].

## 4 NEUROBIOLOGY OF SUDs AND RATIONALE FOR ANTIDEPRESSANT TREATMENT

SUD is a chronic relapsing disorder of the CNS characterized by a phase of binge intoxication followed by a withdrawal syndrome, which reflects the development of dependence, and by a long-lasting phase of vulnerability to relapse, which can be triggered by passive exposure to a substance, spatial cues previously associated with the substance(s), or exposure to stress [129, 178]. The binge phase reflects an abnormal stimulation of the mesolimbic dopaminergic system, which results in an overactivation of both D1 and D2 dopamine receptors in medium spiny neurons of the nucleus accumbens (NAc). Modulation of the mesolimbic system by serotonin is complex and cell- and context-dependent. Activation of 5-HT3 receptors enhances dopamine release in the NAc, whereas 5-HT2A and 5-HT2C receptors differentially modulate dopaminergic neurons in the ventral tegmental area (VTA) [179, 180]. 5-HT2A receptors are preferentially localized on cell bodies of VTA dopaminergic neurons, and their activation stimulates cell firing as a result of intracellular Ca^2+^ mobilization [181]. In addition, activation of cortical 5-HT2A receptors enhances the activity of pyramidal neurons, which send excitatory projection to VTA dopaminergic neurons [182]. In contrast, 5-HT2C receptors are preferentially localized on VTA GABAergic interneurons, and their blockade causes disinhibition of dopaminergic neurons [183]. Nicotinic acetylcholine receptors activate dopaminergic neurons in the VTA and enhance dopamine release from dopaminergic terminals in the NAc [184]. MOR opioid and CB1 cannabinoid receptors positively modulate VTA dopaminergic neurons by restraining the activity of GABAergic interneurons [185-188], whereas KOR receptors, which are endogenously activated by dynorphins, reduce dopamine release from mesolimbic dopaminergic fibers [189, 190]. The withdrawal syndrome is characterized by a reduced mesolimbic dopaminergic transmission, resulting from (i) an overactivation of KOR receptors by endogenous dynorphins [189, 191], (ii) increased activity of neurons of the lateral habenula (“the disappointing center”), which stimulate GABAergic interneurons in the NAc [192], and (iii) a desensitization/downregulation of dopamine receptors in the NAc [193, 194]. The vulnerability phase of the addiction cycle is driven by an enhanced excitatory transmission at the synapse between pyramidal neurons of the prefrontal cortex and medium spiny neurons in the NAc, caused by a reduced activation of presynaptic type-2 metabotropic glutamate receptors, a reduced expression of the glial GLT-1 glutamate transporter, and matrix metalloproteinase-induced degradation of the extracellular matrix, which facilitates the spreading of extracellular glutamate [195, 196].

The multifunctional role of neurotransmitter receptors in the regulation of the mesolimbic system during the “addiction cycle” may drive the choice of antidepressant medication in the comorbidity between MDD and SUD. For example, both SSRIs and SNRIs enhance 5-HT concentrations in the synaptic clef, leading to 5-HT2A, 5-HT2C, and 5-HT3 receptor activation; high doses of fluoxetine may also antagonize 5-HT2C receptors, therefore restraining their inhibitory action on the mesolimbic dopaminergic system [179, 180, 183]. The enhanced noradrenergic transmission induced by SNRIs may support the activation of the mesolimbic dopaminergic system, as shown by the evidence that the ability of cocaine to enhance synaptic dopamine levels is abolished when noradrenaline is selectively depleted in the cerebral cortex [197-199].

The impact of trazodone on the trans-synaptic mechanisms regulating the activity of the mesolimbic system is complex because trazodone is a multifunctional psychotropic drug with dose-dependent effects. Trazodone acts as a sedative-hypnotic medication at low doses, which are sufficient to antagonize 5-HT2A, H1, and α1 receptors, and behaves as an antidepressant at higher doses, which inhibits the high-affinity serotonin transporter (SERT) with a target occupancy >80% (the affinity of trazodone for SERT is 100-fold lower than the affinity for 5-HT2A receptors). Trazodone also behaves as an antagonist of 5-HT2B, 5-HT2C, α1A, α2C receptors and a partial agonist of 5-HT1A receptor [200]. The potent 5-HT2A receptor antagonism by trazodone reduces the risk of adverse effects associated with 5-HT2A receptor stimulation, such as sexual dysfunction, which instead frequently occurs in response to SSRIs. α2 receptor antagonism may play a role in reducing craving and withdrawal symptoms associated with SUD because α2 receptors negatively modulate noradrenaline release [200]. On the other hand, 5-HT2C receptor antagonism could result in lower activity of VTA GABAergic interneurons, causing activation of VTA dopaminergic neurons [183].

Vortioxetine is a multimodal antidepressant acting as a SERT inhibitor, a full agonist of 5-HT1A receptors, a partial agonist of 5-HT1B receptors, and an antagonist of 5-HT1D, 5-HT3, and 5-HT7 receptors [201, 202]. Being a potent 5-HT3 receptor antagonist, vortioxetine is a valuable candidate for the treatment of depression associated with AUD because ethanol positively modulates 5-HT3 receptors [203]. Vortioxetine is also able to improve cognition through the combined antagonism of 5-HT3 and 5-HT7 receptors (see above); this may be highly beneficial in patients affected by SUD because cognitive dysfunction may contribute to the loss of control of substance intake during the three phases of the addiction cycle.

Esketamine has recently been approved worldwide for the treatment of resistant depression and behaves as a potent slow inhibitor of NMDA receptors. This effect may restrain the activity of lateral habenula during the withdrawal syndrome and uncouple the association between substance use and environmental cues. However, ketamine has an established addictive potential, and this raises concern about the use of esketamine for the treatment of depression comorbid with SUD [204-207].

## 5 ANTIDEPRESSANT TREATMENT OF MDD-SUD COMORBIDITY

A valuable approach for the management of patients with MDD-SUD is to treat both disorders at the same time, either with a single drug or a combination of drugs. An integrated treatment for MDD-SUD is more effective than treating the two disorders separately [21, 208, 209]. For example, the combination of sertraline with naltrexone showed greater efficacy in reducing both depressive symptoms and alcohol use compared to individual therapies [22]. Nonetheless, the choice of the most appropriate antidepressants for the treatment of MDD-SUD is difficult, and the clinical outcome is uncertain.

Bupropion was found to be effective in reducing depressive symptoms and substance use (particularly nicotine use) [210, 211], although its use in the treatment of SUD is debated [212, 213]. Previous meta-analyses suggested that the therapeutic efficacy of tricyclic antidepressants (TCAs) and SSRIs in patients affected by MDD-SUD are modest or negligible, and TCAs and SSRIs should always be used in combination with addiction-targeted therapies [76, 211]. In a more recent meta-analysis [214], fluoxetine or sertraline treatment in MDD-SUD adolescents and young adults showed short-term effects on dichotomous indicators of depression but no effect on continuous depression and substance use. In another study, long-term follow-up analysis showed that fluoxetine was effective in academic functioning and AUD and cannabis use disorder, although patients did not achieve full functional recovery, with about 80% showing recurrent episodes of depression [215]. In patients with depression and a history of cannabis use, treatment with fluoxetine, extended-release quetiapine, or extended-release venlafaxine was not superior to placebo [216], perhaps because psychological interventions were more effective than pharmacological treatments, as shown by other studies [76, 217-219]. A Swedish nation-wide register study including almost 150,000 patients with anxiety/depression showed that substance use decreased in response to SSRIs and that the effect of SSRIs depended on the severity of the specific SUD [68]. A recent meta-analysis including 64 randomized trials and over 6,000 participants found that treatment with SSRI improved depressive symptoms, reduced substance craving, and also reduced cocaine and alcohol use, with fluoxetine showing the highest antidepressant effect among SSRIs [220].

Previous reviews and meta-analyses found weak evidence for the benefits of antidepressants in treating symptoms of MDD in patients affected by OUD on OAT [221-224], with TCAs being more effective than SSRIs [221, 224]. This was confirmed by a more recent meta-analysis showing that TCAs were more effective than SSRIs, and SSRIs did not differ from placebo [219]. An important issue is whether and to what extent antidepressants influence OAT retention. In a randomized placebo-controlled trial, Stein *et al*. [225] examined the effect of escitalopram on the retention of buprenorphine treatment in patients affected by OUD and MDD. Buprenorphine improved depressive symptoms on its own, whereas an early treatment with escitalopram did not improve buprenorphine retention and had no additional effect on depressive symptoms. It was concluded that the onset of antidepressant medication should be postponed in patients with OUD under treatment with buprenorphine [225].

In contrast, in a more recent retrospective cohort study including >11,000 individuals, antidepressants administered during OAT improved buprenorphine retention, whereas they increased the risk of buprenorphine discontinuation when given prior to OAT [226]. Buprenorphine was also proposed as an effective therapeutic option for the dual purposes of treating MDD and OUD [227].

Antidepressants lacked efficacy in patients with operationally defined diagnostic criteria for cocaine use disorders [213]. Contingency management, which is a type of therapy in which behaviors are reinforced in close proximity to their occurrence, is highly effective in SUD [228-230]. There is evidence that either TCAs (desipramine) or bupropion act synergistically with contingency management in improving cocaine use disorder [231, 232].

What emerges from all these studies is that data obtained with classical antidepressants, such as TCAs and SSRIs, in the treatment of MDD/SUD, are not homogeneous, and the effect of these drugs is suboptimal and context-dependent. As outlined above, emotional blunting induced by SSRIs and cognitive impairment resulting from the anticholinergic effect of TCAs may be detrimental to SUD and confound the interpretation of clinical studies.

There are only a few studies on the effect of newer antidepressants on individuals affected by MDD/SUD. Intravenous ketamine, which is effective as an antidepressant, is currently being tested for the treatment of SUD (especially AUD) and MDD-SUD comorbidity, but results are still inconsistent [233-235]. The S isomer of ketamine (esketamine), which is delivered by the intranasal route, might be considered a new therapeutic option for patients with treatment-resistant depression comorbid with SUD [117]. However, both ketamine and esketamine behave as slow blockers of NMDA-gated ion channels, and this mechanism might have a strong impact on the neurobiological substrates of SUD. For example, NMDA receptor blockade restrains neuronal activity in the lateral habenula (the “disappointment center”), thus promoting the activation of the mesolimbic dopaminergic system [205].

Vortioxetine, the prototype of multimodal antidepressants, was effective in reducing major depressive episodes independently of the presence of comorbid SUD. Interestingly 71% of patients affected by SUD responded to vortioxetine, whereas only 34% responded to other antidepressants [20]. In a retrospective observational study including 80 MDD-SUD patients, vortioxetine not only reduced depressive symptoms but also improved cognition and functioning. Consumption of all substances, including alcohol, cannabis, and cocaine, decreased substantially in response to vortioxetine treatment [152]. Despite these interesting findings, the study had several limitations, including its retrospective nature, the limited sample size, and the lack of a control group, so that results can not be generalized to different clinical populations. These findings, however, are in line with other real-world studies on MDD alone [149, 236, 237] and MDD-AUD [17].

Finally, it is important to mention the role of non-pharmacological strategies in the treatment of MDD-SUD. Repetitive Transcranial Magnetic Stimulation (rTMS) has shown efficacy in the treatment of depressive disorders comorbid with SUD [238]. rTMS may target brain regions that interconnect mood disorders and drug addiction, such as the dorsolateral prefrontal cortex, improving both depressive symptoms and drug cravings, as shown in patients affected by cocaine use disorder [239]. rTMS treatment can also improve anhedonia [122], which is a key feature of both MDD and SUD.

### 5.1 Antidepressant Treatment of MDD-AUD

Several classes of antidepressants showed efficacy in improving depressive episodes and AUD-related symptoms in patients with MDD-AUD. However, despite the high prevalence and the increased severity of comorbid MDD and AUD, there is a lack of consensus on treatment guidelines. The National Institute for Health and Care Excellence (NICE) and the American Psychiatric Association (APA) guidelines recommend the use of antidepressants only in 3-to-4-week abstinent AUD subjects with co-occurring MDD [7, 240].

**Fig. (1):**
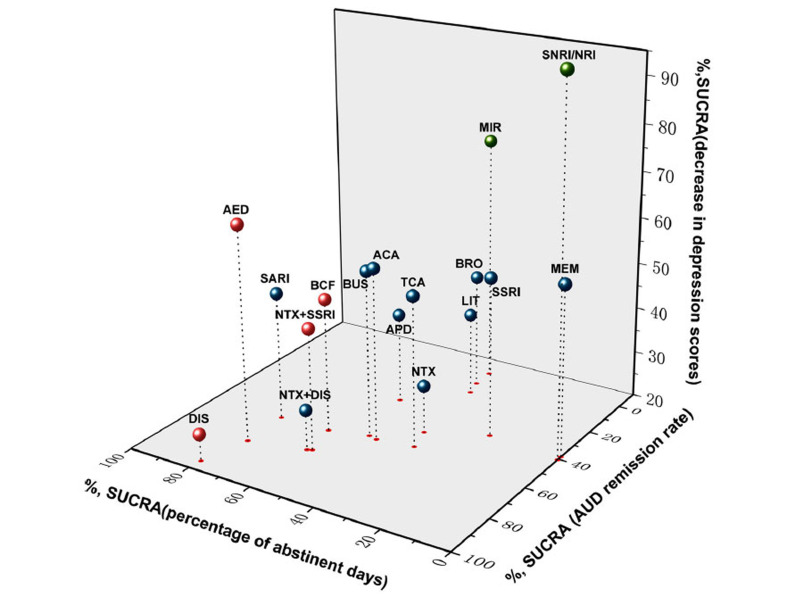
Three-dimensional scatter plot for surface under the cumulative ranking curve (SUCRA) value for three different parameter outcomes. The more a SUCRA value approaches 100%, the better the corresponding intervention is in efficacy. Red colored represented an intervention that was significantly more efficacious in the treatment of alcohol-related symptoms compared to controls. Green-colored represented an intervention that was significantly more efficacious in reducing the scores on the depression scales. **Abbreviations:** ACA, acamprosate; AED, antiepileptics; APD, antipsychotics; BCF, baclofen; BUS, buspirone; BRO, bromocriptine; DIS, disulfiram; LIT, lithium; MEM, memantine; MIR, mirtazapine; NRI, noradrenaline reuptake inhibitor; NTX, naltrexone; SARI, serotonin receptor antagonist/reuptake inhibitor; SSRI, selective serotonin reuptake inhibitor; TCA, tricyclic antidepressants. Reproduced from Li *et al*. [19] and John Wiley & Sons, Inc. under the terms of the Creative Commons Attribution 4.0 International (CC BY 4.0) licence.

To date, investigations on MDD-AUD treatment have focused mainly on the effects of SSRIs, while other antidepressants have been less investigated. TCAs and nefazodone were more effective than placebo in treating depressive symptoms [241]. Trazodone was a valuable option in treating insomnia during alcohol withdrawal, with no cross-tolerance with alcohol, unlike benzodiazepines [242]. The extended-release formulation of trazodone reduced depressive symptoms, anxiety, sleep alterations, and craving in patients with MDD-AUD [18]. Systematic reviews and meta-analyses showed that several classes of antidepressants can improve MDD and some AUD symptoms, such as the number of drinking days and the rate of abstinent patients [243-245]. Monotherapy, either involving anti-craving drugs or antidepressants, may not be sufficient to improve both MDD and AUD symptoms [19, 246]. For example, treatment with SSRIs and TCAs improved MDD symptoms bur was less effective in reducing AUD-related symptoms [246]. In the Bayesan meta-analysis by Li *et al*. [19], disulfiram showed the best efficacy in achieving remission from AUD; noradrenaline reuptake inhibitors substantially reduced depressive symptoms, whereas antiepileptic drugs had an effect on both MDD and AUD (Fig. **1**). The US veteran administration recommends topiramate as the first treatment option for AUD [247]. However, in a recent meta-analysis, fluoxetine improved both depressive symptoms and AUD regardless of treatment length or dosage [220]. Other studies confirmed the ability of SSRIs (in particular, fluvoxamine, fluoxetine, and citalopram) to improve depressive symptoms and alcohol craving [248-251].

Newer antidepressants are also being tested in MDD-AUD patients. Ketamine, administered in combination with naltrexone, improved depressive and AUD-related symptoms in a small number of patients [235].

Two real-world studies demonstrate the efficacy of vortioxetine in reducing alcohol consumption and improving depressive symptoms [17, 152]. The retrospective study by Di Nicola *et al*. [17] included 56 MDD and 57 MDD-AUD outpatients matched for baseline characteristics and psychiatric comorbidities. Vortioxetine treatment was safe and well tolerated and improved depressive symptoms to the same extent in the two groups of patients, suggesting that AUD did not affect the therapeutic efficacy of vortioxetine. Vortioxetine has the potential to reduce alcohol cravings because it behaves as a potent antagonist of 5-HT3 receptors (see 4. Neurobiology of SUDs and rationale for Antidepressant treatment). Activation of 5-HT3 by ethanol contributes to alcohol craving by stimulating the mesolimbic dopaminergic system. KOR opioid receptors can also be targeted by pharmacological intervention in AUD and MDD-AUD. Nalmefene, a partial agonist/antagonist of KOR receptors, is currently indicated for the reduction of alcohol consumption in adult patients with AUD [189, 190, 252, 253]. Aticaprant, a selective KOR antagonist, is under development for the treatment of MDD [254].

## 6 EXPERT OPINION

MDD and SUD affect each other bidirectionally, resulting in worse overall symptomatology and increased risk of death (Table **2**) [1, 8]. Thus, extra care should be taken when treating these patients, and the two conditions should be treated concurrently [21, 255, 256]. Despite this, MDD-SUD patients are undertreated in the current clinical practice, and this has a strong socioeconomic impact because of inadequate treatment and continued need for care [70]. Special efforts should be undertaken by stakeholders and healthcare professionals to ensure proper treatments for patients affected by MDD-SUD. Large RCTs examining the efficacy of individual antidepressants on depressive and SUD-related symptoms are urgently needed.

It is fundamental to personalize treatment and understand which intervention is the most effective according to patients’ preferences and characteristics, MDD subtype, specific SUD, and long-term effects. Both MDD and SUD cause detrimental effects on psychosocial, financial, and occupational functioning, which, in turn, affect the overall QoL and other patient-reported outcomes. From this perspective, gaining deeper insights into clinical variables and multifaceted dimensions typical of MDD-SUD comorbidity could pave the way for targeted treatments, potentially enhancing treatment response. Anhedonia and impaired cognition should be targeted by effective treatments in MDD-SUD [100, 257] (see section 3.2 Focus on specific clinical features of MDD-SUD). Additionally, treatment-related effects that could worsen SUD, such as SSRI/SNRI-induced emotional blunting, should be taken into consideration [135, 258]. Cognitive and reward brain circuits are highly interconnected, and cognitive function is pivotal in controlling substance use and managing craving [137, 139]. Anhedonia plays a key role in the development and co-occurrence of MDD and SUD, is associated with craving and novelty-seeking, and is particularly pronounced during the detoxification phase of SUD patients [100]. In the case of comorbid OUD, it is important to deliver OAT and monitor if and how antidepressant treatment influences OAT retention.

In light of these considerations, antidepressant medications acting on cognitive functions, craving, and anhedonia could be particularly valuable in the treatment of MDD-SUD.

Vortioxetine has the potential to be an effective drug in MDD-SUD because it is the antidepressant with the greatest efficacy on cognitive dysfunction associated with MDD owing to 5-HT3 and 5-HT7 receptor blockade [101, 135, 141, 148, 152, 257]. Vortioxetine has also shown efficacy in improving anhedonia and social functioning in patients affected by MDD [121, 135]. Observational and interventional studies have shown that vortioxetine significantly improved functional outcomes inpatients affected by MDD and MDD-SUD [17, 102, 135, 152, 257]. Moreover, vortioxetine could be effective in treating MDD patients with partial response to SSRI and SNRI and improve the SSRI/SNRI-induced emotional blunting [135]. Vortioxetine fails to increase dopamine release in the nucleus accumbens and, therefore, has no addictive potential [259]. As opposed to other antidepressants (*i.e*., fluoxetine, paroxetine, fluvoxamine, duloxetine, venlafaxine, and bupropion), vortioxetine does not inhibit drug metabolism, and a limited drug-drug interaction might be an added value in a context of polytherapy or poly-substance use [260, 261]. Alternative therapeutic options include (i) trazodone, owing to its efficacy in improving sleep disturbances without inducing emotional blunting and with a favorable adverse event profile; (ii) esketamine, owing to its pro-hedonic effect in treatment-resistant depression (concerns remain on the potential addictive potential of esketamine); and (iii) rTMS, for its rapid onset of action, lack of pharmacodynamic interactions, the potential impact on craving, and pro-hedonic effects.

## CONCLUSION

Depressive and substance use disorders are highly intertwined, with AUD being the most common SUD occurring in MDD patients. Individuals with a dual diagnosis show more severe depressive symptoms, greater functional impairment, a more chronic course of illness, lower rates of recovery, higher rates of hospitalization, and increased morbidity and mortality and risk of death. Specific clinical features of MDD-SUD patients include increased levels of anhedonia and cognitive dysfunction, which often result in the inability to control craving and relapses, and reduced adherence to treatment, establishing a vicious circle in which SUD precipitates MDD and *vice versa*. For these reasons, patients with psychiatric disorders and comorbid SUD should be treated with extra care; however, they receive insufficient treatment because of the lack of reliable guidelines.

It is not clear to what extent patients with MDD-SUD benefit from antidepressant treatment. Data from the literature are inconclusive and often conflicting. This is due to general difficulties in carrying out RCTs and the fact that patients with comorbid SUD are often excluded from trials investigating MDD treatments, resulting in a lack of high-quality evidence.

The choice of a specific drug should be tailored to the patient’s clinical history and expectations, considering the specific SUD and MDD subtypes. Ideal medications for MDD-SUD should act on anhedonia, craving, and cognitive function, avoid emotional blunting, not interact with other drugs, and have no addictive potential.

## Figures and Tables

**Table 1:** MDD and SUD diagnostic criteria according to the DSM-5.

**MDD Symptoms**	**SUD Symptoms**
1. Depressed mood or a loss of interest or pleasure in daily activities. 2. Significant weight loss or gain or changes in appetite. 3. Insomnia or hypersomnia (excessive sleeping). 4. Psychomotor agitation or retardation (e.g., restlessness or slowed movements). 5. Fatigue or loss of energy. 6. Feelings of worthlessness or excessive guilt. 7. Diminished ability to think or concentrate, or indecisiveness. 8. Recurrent thoughts of death, suicidal ideation, or a suicide attempt.	1. Taking the substance in larger amounts or for longer than intended. 2. Wanting to cut down or quit but not being able to do so. 3. Spending a lot of time getting, using, or recovering from the substance. 4. Cravings and urges to use the substance. 5. Failing to meet major obligations at work, school, or home due to substance use. 6. Continuing to use the substance despite persistent or recurrent social or interpersonal problems caused or exacerbated by the effects of the substance. 7. Giving up or reducing important social, occupational, or recreational activities because of substance use. 8. Using the substance in situations where it is physically hazardous. 9. Continuing to use the substance despite knowledge of having a persistent or recurrent physical or psychological problem that is likely to have been caused or exacerbated by the substance. 10. Tolerance as defined by either a need for markedly increased amounts of the substance to achieve intoxication or desired effect or markedly diminished effect with continued use of the same amount of the substance. 11. Withdrawal, as manifested by either the characteristic withdrawal syndrome for the substance or the substance is taken to relieve or avoid withdrawal symptoms.
To receive a diagnosis of MDD, a person must experience at least five of these symptoms during the same 2-week period, and at least one of the symptoms must be either depressed mood or loss of interest or pleasure. The symptoms must also cause significant distress or impairment in social, occupational, or other important areas of functioning.	In order to meet the diagnostic criteria for SUD, an individual must have at least two of these symptoms within a 12-month period. The severity of SUD is determined by the number of symptoms an individual displays, with mild SUD being diagnosed when an individual displays 2-3 symptoms, moderate SUD when an individual displays 4-5 symptoms, and severe SUD when an individual displays 6 or more symptoms.
DSM-5, Diagnostic and Statistical Manual of Mental Disorders, Fifth Edition. MDD, major depressive disorder. SUD, substance use disorder.	

**Table 2:** Differences between patients with MDD-SUD and patients with MDD only, according to Blanco *et al*. [8].

-	MDD-SUD *vs*. MDD
Family history of MDD, SUD, and antisocial behavior	**↑**
Childhood risk factors (parental loss by separation and early-onset anxiety disorder)	**↑**
Adulthood risk factors (low emotional reactivity, divorce, and stressful life events)	**↑**
The number of DSM-IV MDD criteria met	**↑**
Depressed mood	**↓**
Loss of interest	**↑**
Weight loss/gain	**↔**
Insomnia/hypersomnia	**↑**
Retardation	**↔**
Fatigue	**↔**
Feelings of worthlessness	**↑**
Trouble concentrating	**↑**
Thoughts of death	**↑**
Suicide attempts	**↑**
Age at MDD onset	Earlier
Number of depressive episodes	**↑**
Duration of MDD	**↔**
Psychiatric comorbidities (any axis I and axis II diagnosis)	**↑**
Dysthymia	**↑**
Any anxiety disorder	**↑**
Panic disorder	**↑**
Social anxiety disorder	**↑**
Specific phobia	**↑**
Generalized anxiety disorder	**↔**
Conduct disorder	**↑**
Pathological gambling	**↑**
Psychotic disorder	**↑**
Any personality disorder	**↑**
Avoidant	**↔**
Dependent	**↓**
Obsessive-compulsive	**↑**
Paranoid	**↑**
Schizoid	**↑**
Histrionic	**↑**
Antisocial	**↑↑**
likeliness to receive outpatient treatment	**↔**
likeliness to receive inpatient treatment	**↑**
Alcohol/drug use to relieve depressive symptoms	**↑↑**
Nicotine dependence	**↑↑**

**Abbreviations:** MDD, Major Depressive Disorder. SUD, Substance Use Disorder. ↑, Higher. ↑↑, Much Higher. ↓, Lower. ↔, Same.

## LIST OF ABBREVIATIONS

AUD: Alcohol Use Disorder
MDD: Major Depressive Disorder
OUD: Opioid Use Disorder
rTMS: Repetitive Transcranial Magnetic Stimulation
SSRIs: Selective Serotonin Reuptake Inhibitors
SUD: Substance Use Disorder
TCAs: Tricyclic Antidepressants
